# Can the Federal Baldrige Survey Measure Workforce Well-being in an Academic Health Center?

**Published:** 2019-02-26

**Authors:** Robert Badgett, Jiatian Chen, Douglas R. May, Tom Field, K. Allen Greiner

**Affiliations:** 1 University of Kansas School of Medicine-Wichita, Department of Internal Medicine; 2 University of Kansas School of Business, Lawrence, KS; 3 University of Kansas Medical Center Organizational Improvement Office, Kansas City, KS; 4 University of Kansas School of Medicine-Kansas City, Department of Family Medicine

**Keywords:** academic medical centers, professional burnout, workplace, health promotion, surveys and questionnaires

## Abstract

**Introduction:**

Experts suggest health care institutions switch focus from measuring burnout to measuring positive organizational psychology. Concerns include burnout being a late sign of organizational decline. The Baldrige survey is promoted by the U.S. Department of Commerce to measure positive worksite conditions (e.g., workforce wellbeing of industries, including health care and education). For years, the survey has been completed by managers within organizations, but now the same survey is promoted for completion by an organization’s workforce. We tested the structure of the Baldrige survey when completed by an academic health care workforce. In addition, we tested whether the results in an academic worksite correlate with an example metric of an organizational mission.

**Methods:**

In 2015, our academic health center surveyed faculty and staff with the Baldrige survey. The validity of the Baldrige was tested with confirmatory factor analyses. Within the School of Medicine, responses for the Baldrige’s concepts were correlated against a measure of organizational outcome: graduates’ assessments of Departmental educational quality.

**Results:**

The structure of the Baldrige survey did not validate when assessed by a workforce (RMSEA = 0.086; CFI = 0.829; TLI = 0.815). None of its concepts correlated with learner reported educational quality.

**Conclusions:**

The Baldrige survey, when administered to a workforce rather than managers, did not appear to measure workforce well-being within an academic health care center. We discourage use of the current survey for this purpose.

## INTRODUCTION

The well-being of physicians in the United States, as measured by rates of burnout, is declining.^1^ Accordingly, the well-being of health care personnel has been proposed as a fourth aim of health care in addition to the health of a population, the patient experience of care, and the cost of care.^2^,^3^ The health care system’s focus on burnout, rather than positive organizational psychology, has been questioned.^4^,^5^ Burnout is likely an end state in organizational decline, so focusing on burnout may delay detection of organizational dysfunction.

The Baldrige survey is an excellent candidate for measuring positive organizational health and has been used by industries, including healthcare and education.^6^ The Baldrige framework and criteria for performance excellence was created by the Malcolm Baldrige National Quality Improvement Act of 1987 and is managed and its use encouraged by the National Institute of Standards and Technology (NIST) within the U.S. Department of Commerce. The survey and national benchmarks are freely available. The Baldrige institutional assessment includes a questionnaire that measures seven concepts of organizational tactics. While the survey was originally intended to be completed by managers within an organization applying for the Malcolm Baldrige National Quality Award, administration of the survey to front-line personnel is now encouraged by the NIST.^7^ Although the survey is encouraged for front-line personnel by the NIST, the survey’s structure has not been validated for this purpose. The objective of this study was to assess the structural validity of the Baldrige questionnaire when front-line personnel of an organization are queried. In addition, we correlated the concepts within the Baldrige with a measurement of an institutional goal.

## METHODS

### Study design and setting

An analysis of existing, anonymous data that had been collected for operational purposes by the Organizational Improvement Office (OIO) at the University of Kansas Medical Center was performed.

### Participants

Invitations to the online, anonymous survey were emailed by OIO to all faculty and staff in 2015. One reminder email was sent.

### Measurements

We used the Baldrige “Are We Making Progress” 2011 questionnaire (Table 1). The questionnaire contains 40 Likert questions that query the presence of four to nine positive attributes per seven concepts (leadership, strategy, customer focus, workforce focus, information management, operations, and results).

The organizational outcome of interest was educational quality which was measured using the ratings of clinical clerkships by recent graduates of the school. The ratings were gathered from the 2014 Association of American Medical Colleges graduation questionnaire (AAMC-GQ).^8^ The AAMC-GQ provides separate ratings for clerkships in internal medicine, family medicine, surgery, pediatrics, psychiatry, and obstetrics and gynecology. The ratings were normalized by using the national percentile ratings for each clerkship rather than use the raw ratings by our graduates.

### Statistical methods

A confirmatory factor analysis (CFA) validated the structure of the Baldrige questionnaire. Statistical fit of the model was evaluated with the root mean square error of approximation (RMSEA), comparative fit index (CFI), and the Tucker-Lewis index (TLI). Acceptable fit is indicated by RMSEA less than 0.06, CFI above 0.90, and TLI above 0.90.^9^ Analyses were done with the Lavaan Package for R Programming Language.^10^

For departments or divisions that sponsor a clinical clerkship within the School of Medicine, mean values of responses were calculated by personnel within the work unit to each item within the seven Baldrige concepts. Then, a mean for each Baldrige concept was calculated. The means of the responses were correlated for each concept to graduates’ assessments of departmental educational quality. Calculations were done with the R Programming Language.^11^

## RESULTS

Responses were received from 877 faculty and staff for a response rate of 21%. The Baldrige did not validate by confirmatory factor analysis with all measures of fit not meeting thresholds for validity (RMSEA = 0.086; CFI = 0.829; TLI = 0.815).

None of the seven concepts of the Baldrige as assessed by departmental personnel significantly correlated with educational quality as assessed by recent graduates (Table 2). The range of correlation coefficients ranged from −0.01 for the concept of “Results” to 0.58 for the concept of “Customer Focus”.

## DISCUSSION

Our academic health center, which like all academic health centers combines both delivery of health care and provision of higher education, did not validate the structure of the Baldrige for measuring its seven concepts. In addition, the Baldrige concepts did not correlate with our measure of an organizational goal, the AAMC-GQ. The negative results may reflect that most prior studies of the Baldrige queried managers and external assessors of organizations rather than front-line personnel.^12^–^22^ In addition, most of these studies created custom surveys based on Baldrige concepts.^13^,^14^,^16^,^20^,^22^

Four previous studies attempted validation of the original Baldridge questions.^12^,^15^,^21^ Two studies surveyed leaders or managers in diverse industries and were able to validate the survey after modifying the structure.^12^,^15^ In a third study, Jayamaha et al.^21^ surveyed Baldrige personnel who formally assessed companies that applied for the Baldrige award. They found low discriminant validity suggesting questions belonged to multiple concepts. The only study that surveyed frontline personnel, like our study, was not able to validate the relationships between concepts of the Baldrige model.^23^

Workforce conditions should move beyond current recommendations to measure burnout and instead measure positive organizational psychology.^4^,^5^ Unfortunately, the Baldrige does not support this goal. Other studies support the concept of thriving (defined as a workforce that is both engaged and learning or improving) should be measured.^24^,^25^ Thriving has been studied in industries other than health care and found to correlate with job performance of both individuals and groups.^24^,^25^ On the other hand, burnout, while correlated with quality of care as perceived by physicians,^26^ did not correlate with measured quality of care in the “Minimizing Error, Maximizing Outcome” study^27^ or Healthy Work Place trial.^28^ In addition, as previously noted, focusing on burnout may delay detection of organizational dysfunction. In addition to helping academic health centers meet organizational goals, successful measurement of workforce well-being may have larger impact by using controlled, public reporting to address physician burnout in clinical practice.^3^

Our study is limited by a low response rate. However, this rate is typical of national studies of burnout.^1^ With a larger study size, our correlation of ‘customer focus’ of departments with recent graduates’ satisfaction with departmental clerkships might become statistically significant. However, even if this correlation is significant, the structure of the Baldrige does not validate and better surveys should be sought. In 2015, the Baldrige survey was revised; however, only a single question was reworded.

Our negative finding regarding the Baldrige’s inability to measure the perspective of front-line personnel should not be generalized to other roles of the Baldrige framework and awards. For example, other studies using external examiners show receipt of the Baldrige award in health care correlates with organizational financial performance^29^ and positive experiences by patients.^30^ However, as noted previously, the only prior study that attempted validation of the structure of the Baldrige survey to measure the perspective of front-line personnel using the original questions in the survey was also negative.

## CONCLUSIONS

We discourage use of the Baldrige survey to measure employee perceptions of well-being in academic health centers. The lack of validation studies of front-line personnel in any industry questions the use of the Baldridge by frontline personnel in any setting. Our negative findings are important as workforce well-being is an emerging issue and the NIST is promoting the Baldrige “Are we making progress” survey for measuring the front-line perspective.

## Figures and Tables

**Table 1:** The Baldrige “Are We Making Progress” 2011 questionnaire.

Baldrige Concept	Questions
Leadership	I know my organization’s mission (what it is trying to accomplish). I know my organization’s vision (where it is trying to go in the future). My senior (top) leaders use our organization’s values to guide us. My senior leaders create a work environment that helps me do my job. My organization’s leaders share information about the organization. My organization asks what I think.
Strategic planning	As it plans for the future, my organization asks for my ideas. My organization encourages totally new ideas (innovation). I know the parts of my organization’s plans that will affect me and my work. I know how to tell if we are making progress on my work group’s part of the plan. My organization is flexible and can make changes quickly when needed.
Customer focus	I know who my most important cutomers are. I regularly ask my customers what they need and want. I ask if my customers are satisfied or dissatisfied with my work. I am allowed to make decisions to solve problems for my customers. I also know who my organization’s most important customers are.
Measurement, analysis, and knowledge management	I know how to measure the quality of my work. I can use this information to make changes that will improve my work. I know how the measures I use in my work fit into the organization’s overall measures of improvement. I get all the important information I need to do my work. I know how my organization as a whole is doing.
Workforce focus	The people I work with cooperate an work as a team. My bosses encourage me to develop my job skills so I can advance in my career. I am recognized for my work. I have a safe workplace. My bosses and my organization care about me. I am committed to my organization’s success.
Operations focus	I can get everything I need to do my job. We have good process for doing our work. I have control over my work processes. We are prepared to handle an emergency.
Results	My work products meet all requirements. My custormers are satisfied with my work. I know how well my organization is doing financially. My organization has the right people and skills to do its work. My organization removes things that get in the way of progress. My organization obeys laws and regulations. My organization practices high standards and ethics. My organization helps me help my community. My organization is a good place to work.

**Table 2:** Correlations of the seven concepts of the Baldrige as assessed by departmental personnel with educational quality as assessed by recent graduates.

Baldrige Concept	Correlation with recent graduates’ satisfaction with departmental clerkships	p - value
Leadership	0.12	0.72
Strategic planning	0.138	0.71
Customer focus	0.58	0.06
Measurement, analysis and knowledge management	0.40	0.22
Workforce focus	0.07	0.83
Operations focus	0.31	0.37
Results	−0.01	0.98
